# Enabling and hindering aspects of the i²TransHealth e-health intervention for transgender and gender diverse people in Germany: a qualitative process evaluation

**DOI:** 10.1186/s12913-026-14026-y

**Published:** 2026-01-24

**Authors:** Franziska Schmidt, Janis Renner, Samantha-Insine Schröder, Lea Pregartbauer, Arne Dekker, Timo O. Nieder

**Affiliations:** Institute for Sex Research, Sexual Medicine and Forensic Psychiatry, University Medical Centre Hamburg-Eppendorf, Martinistraße 52, 20246 Hamburg, Germany

**Keywords:** RCT, Process evaluation, Qualitative text analysis, Implementation, e-health, Digital health, Transgender, Gender diverse, Remote care, Barriers to accessing healthcare

## Abstract

**Background:**

Transgender and gender diverse (TGD) people in remote areas face structural barriers to accessing gender-affirming, interdisciplinary healthcare. These include limited specialised care, a shortage of TGD-informed healthcare professionals, long travel distances to urban care centres, and insufficient local crisis support. The i²TransHealth intervention addressed these gaps by offering video consultations, chat-based support, and a network of locally trained physicians (general practitioners, psychiatrists). This qualitative process evaluation identified enabling and hindering aspects to inform recommendations for improving and scaling i²TransHealth, and to support the implementation of e-health in other TGD-informed care contexts.

**Methods:**

i²TransHealth was evaluated as an e-health intervention in a randomised controlled trial involving 174 TGD adult participants (aged 18 and above) from northern Germany. As part of the intervention group, service users received video consultations and chat-based support provided by study therapists, along with outpatient care from general practitioners and psychiatrists as needed. To capture the experiences of those involved in delivering and receiving the intervention, four online focus groups were conducted with service users (*n* = 4), study therapists (*n* = 5), general practitioners (*n* = 6), and psychiatrists (*n* = 7). Data were collected using semi-structured interview guides and analysed according to Kuckartz’s thematic text analysis.

**Results:**

The process evaluation identified enabling and hindering aspects influencing the implementation of i²TransHealth. Focus group participants reported improved access to TGD-informed healthcare through i²TransHealth, though this was challenged by factors such as time constraints, administrative burdens, and limited referral options for specialised care. They highlighted the flexibility and support of study therapists, as well as reduced distress and increased wellbeing of service users. While video consultations facilitated access, they were considered challenging for assessing non-verbal cues and treating highly distressed individuals. The e-health platform enabled easily accessible communication, yet usability issues, inconsistent engagement, and high workload for study therapists were noted.

**Conclusions:**

E-health interventions like i²TransHealth can enhance access to TGD-informed healthcare in remote areas, but require integration with in-person services for complex cases involving highly distressed individuals. Expanding training, strengthening networks, and refining digital tools are crucial for sustainability.

**Trial registration:**

The trial was registered in ClinicalTrials.gov 28/02/2020 (NCT04290286).

**Supplementary Information:**

The online version contains supplementary material available at 10.1186/s12913-026-14026-y.

## Background

Transgender and gender diverse (TGD) people from remote areas face specific structural disadvantages in accessing and maintaining TGD-informed healthcare. They face long journeys to access specialised and interdisciplinary care, in addition to the long waiting times that apply to all TGD people seeking care [1–4]. Some must coordinate weekly or monthly appointments at distant clinics, which can increase psychological distress due to frequent absences from education or work [5–7]. Furthermore, access can be impeded by local, non-specialised or dismissive healthcare professionals (HCPs) [8–11]. Beyond logistical challenges, restricted access to TGD-informed healthcare in remote areas may contribute to adverse health outcomes, including heightened mental health vulnerability and delayed or interrupted gender-affirming medical treatments [2]. Throughout this article, ‘remote’ refers to geographically isolated regions lacking TGD-informed healthcare, with specific aspects of rural socialisation addressed where relevant.

The e-health intervention i²TransHealth, conducted by the University Medical Centre Hamburg-Eppendorf (UKE), Germany, was developed to reach TGD people in underserved remote areas early in their transition or gender exploration. In the absence of accessible, TGD-informed local services, the e-health intervention aimed to provide psychosocial support and assess individual healthcare needs. By delivering targeted information, counselling, and care, i²TransHealth sought to overcome the previously mentioned structural barriers [12–15].

i²TransHealth, an acronym for an interdisciplinary, internet-based intervention for TGD people, was tested using several evaluation methods [16]. These included a randomised controlled trial (RCT) with 174 TGD adult participants (aged 18+), in which the intervention served as the experimental condition compared to a wait-list control, an economic evaluation of cost-effectiveness and excess costs, and a quantitative and qualitative process evaluation [16]. This article focuses on the qualitative part of the process evaluation, which investigates enabling and hindering aspects in the implementation of i²TransHealth.

As an e-health intervention, i²TransHealth consisted of video consultations and 1:1 chat conversations with trained study therapists. In accordance with German telehealth regulations, each TGD participant had an initial in-person appointment with a study therapist prior to randomisation, and all subsequent interactions during the intervention phase took place online. After completion of the RCT, ongoing care continued primarily online, supplemented by quarterly in-person appointments. The intervention provided a low-threshold (easily accessible) entry point to discuss transition-related concerns, clarify care needs, and rebuild trust in the healthcare system. In addition, i²TransHealth collaborated with a local network of physicians in northern Germany, established as part of the e-health intervention, to ensure crisis intervention close to the participants’ homes when needed. Online communication between study therapists and service users, as well as between the study team and network physicians, took place via a secure access area of an e-health platform. The publicly accessible part of the e-health platform served as the official website and gateway to the service.

The study therapists were TGD-informed mental health professionals who offered remote online support to service users. These service users consisted exclusively of study participants from the intervention group of the RCT. They were invited to use i²TransHealth for four months, with the option to continue care thereafter, either online or on-site. Participants assigned to the control group waited four months before gaining access to the e-health intervention or starting standard on-site care. During the video consultations held every two weeks, study therapists conducted diagnostics, gender-affirmative counselling, and care planning, and, if necessary, provided mental health support (e.g., short-term psychotherapy). In addition, between video consultations, study participants could contact their study therapists via a chat function and share documents with them (e.g., medical examination letters). The public i²TransHealth website (www.i2transhealth.de) featured an overview of frequently asked questions (FAQs), study framework conditions, details on the available care, transition- and TGD-related information, updates on relevant topics, and a list of the local physicians involved in the i²TransHealth network.

Information and communication technologies (ICT) do not replace in-person interactions, as face-to-face treatments (e.g., physical examinations) remain indispensable for patients in the long term and enable a wider range of treatments within an integrative blended approach [17–21]. For this reason, we implemented a local physicians’ network, in which both a primary care and a psychiatric care practice were involved at each of six locations spread across the northern German federal states of Bremen, Mecklenburg-Western Pomerania, Lower Saxony and Schleswig-Holstein. They received training on the specific healthcare needs of TGD people during a two-day kick-off event, and remained in contact with the study coordination team throughout the study period via online round tables and an online forum (within the protected area of the e-health platform). Accordingly, the intervention involved two main groups of HCPs: (1) study therapists, who provided TGD-informed psychosocial support via video consultations and 1:1 chat conversations; and (2) local network physicians, including general practitioners (GPs) and psychiatrists, who offered optional in-person medical support if needed. While study therapists were the primary contact for service users, the network physicians were available as an optional local resource to address physical health or psychiatric concerns.

Previous research suggests that e-health services can be a useful addition to TGD-informed healthcare [2, 12, 22, 23]. In this text, we provide an in-depth understanding of the experiences of delivery staff and participants based on the qualitative process evaluation. Focus groups with study therapists, network physicians, and service users allowed us to analyse the enabling and hindering aspects of the components, processes, and surrounding conditions of i²TransHealth. Complementing the statistical analysis of the effectiveness of i²TransHealth [24, 25] and the health economic evaluations [26, 27], the qualitative process evaluation provides insights into how the intervention works.

## Methods

### Aim

The aim of the qualitative process evaluation was to analyse the aspects that enabled or hindered the delivery of the i²TransHealth intervention, as experienced by its HCPs and service users. This analysis was intended to provide a comprehensive understanding of how the intervention was implemented in practice. Based on the findings, the evaluation sought to develop recommendations for the improvement, continuation, and expansion of i²TransHealth, as well as to generate transferable guidance for TGD-informed e-health interventions more broadly.

### Design of the process evaluation

The process evaluation was designed to gain an in-depth understanding of the e-health intervention, the local physician network, and the e-health platform. Qualitative methods were chosen to allow for reflection on practical adaptations, ineffective elements, and unexpected effects [28]. The sample included the study therapists, the network physicians, and a selection of service users as the key actors of i²TransHealth. They were interviewed in focus groups in the second year of the i²TransHealth intervention. This timing was selected based on the assumption that study therapists and network physicians would have gained substantial experience with the intervention by that point. Furthermore, it was expected that a group of service users who had completed the four-month RCT intervention period would be available and sufficiently familiar with i²TransHealth to provide in-depth reflections. The process evaluation was carried out as an internal evaluation by the study coordination team (TN, AD), research and clinical staff (JR, LP, SS), as well as a researcher (FS) from the same organisation who was not involved in the study.

### Participants and setting

All study therapists and network physicians involved in the i²TransHealth intervention were invited to participate in the focus groups. Service users were selected and recruited with the aim of including individuals with diverse gender identities, assigned sexes at birth, ages, and places of residence at the start of the e-health intervention, with the intention of forming a balanced and representative group for the qualitative evaluation. As the RCT was still ongoing at the time of the qualitative process evaluation, only service users who had completed the four-month intervention were eligible. Four separate focus groups were conducted: one with service users, one with study therapists, and two with network physicians (one with GPs and the other with psychiatrists). All participants were invited to take part in focus groups held via video conference.

### Data collection procedures

The focus groups were conducted via video conference using a semi-structured interview guide (cf. supplementary material), which was developed in several stages following the recommendations of Helfferich [29]. With regard to the structure of i²TransHealth, it included the following thematic blocks: open thematic entry, concrete care experiences, experiences with the i²TransHealth network and infrastructure, and special events. Each thematic block was mapped using one to four guiding questions. The introductory questions were asked in all focus groups; follow-up questions were asked and adapted according to emerging themes and group dynamics. At the beginning, the participants were encouraged to engage in conversation with each other.

The interviewees received detailed information about participation, and informed consent was obtained from all participants in writing. The four focus groups took place on 25 August (GPs, part of the network physicians group), 1 September (psychiatrists^1^, also part of the network physicians group), 15 September (study therapists), and 6 October 2021 (service users), respectively, each lasting an average of 90 min. They were conducted by the study coordination team (TN, AD) and the scientific staff (JR). In the focus group with the study therapists, TN was absent due to his role as clinical lead in i²TransHealth. In the focus group with the service users, AD was unable to attend due to scheduling constraints. The focus groups were audio recorded, transcribed verbatim, and pseudonymised. No video recordings were obtained.

### Data analysis

The transcripts were analysed using Kuckartz’s thematic text analysis [30, 31], which advises researchers to first roughly structure the material into main categories and then further differentiate it into subcategories. We began by familiarising ourselves with the material and writing short case summaries. It became clear that i²TransHealth was perceived differently both within and between the focus groups. We therefore decided to use a single overarching system of categories that would allow us to present similarities and differences in a structured and comparative way. The main categories were derived from the interview guide, and the material was roughly structured in the first round of coding. During this process, the main categories were refined, and previously uncovered topics were incorporated. In the second step, subcategories were developed inductively. To achieve this, all text passages were grouped by main category, relevant dimensions were identified, and subcategories were iteratively refined. The entire data set was then coded using the subcategories. The coding process was conducted using the Atlas.ti software package (version 8.4.5). Two coders (FS, JR) divided the data, cross-checked the codes, and resolved disagreements through consultation until consensus was reached. The structure and components of the category system were discussed and agreed upon by all authors. The analysis and writing of the report followed the main categories. This involved arranging the subcategories in a meaningful order and analysing the similarities, differences, and characteristics of each subcategory.

## Ethical considerations

The study was conducted according to the Declaration of Helsinki and approved by the Hamburg Medical Association (PV7131). The study was submitted to the National Institutes of Health Clinical Trials on 28/02/2020 (www.clinicaltrials.gov/; Identifier: NCT04290286).

## Results

### Sample

The key stakeholder groups involved in the i²TransHealth intervention were well represented in the focus groups. Service users (*n* = 4) shared their experiences of engaging with the intervention as TGD people. Study therapists (*n* = 5), GPs (*n* = 6), and psychiatrists (*n* = 7) offered professional perspectives informed by varying degrees of prior experience with TGD people. The different groups of interviewees had the following characteristics:

*Service users*: Four service users were interviewed (age: M = 30.3, SD = 11.6, range 21–47). The group included one trans woman, one trans man, one genderfluid trans feminine person, and one genderfluid genderqueer non-binary person. There was an equal distribution of sex assigned at birth. Participants lived in different locations at the start of the e-health intervention: one in a small town, one in a medium-sized town, and two in large towns.

*Study therapists*: Five out of seven study therapists at the UKE participated in the focus groups. Of these, four were cis women and one was gender-expansive (age: M = 32.8, SD = 5.9, range 27–42). They had been providing mental health support to TGD people in their psychotherapeutic work for an average of 1.7 years (SD = 1.2, range 0.5–3). Study therapists were fully qualified psychologists who had completed a diploma or master’s degree and were undergoing advanced clinical training as part of the German state licensing pathway to become psychotherapists.

*Network physicians*: The GP group consisted of two cis women and four cis men (age: M = 49.0, SD = 4.6, range 42–54) who had been working with TGD people in their practice for an average of 7.6 years (SD = 2.2, range 6–10). One GP chose not to disclose their age or level of experience with TGD patients. The psychiatrist group consisted of four cis women and three were cis men (age: M = 53.3, SD = 6.6, range 41–61) who had been working with TGD people in their practice for an average of 13.0 years (SD = 11.6, range 1–35). Some network physicians – both GPs and psychiatrists – stated that they had only begun engaging intensively with TGD patients in their practice since the start of the e-health intervention, and in some cases had rarely treated TGD patients before.

### Thematic categories from the focus group analysis

The experiences shared and discussed in the focus groups were organised into six main categories, each further divided into three to five subcategories (cf. Table 1). These categories capture the essential elements, processes, and surrounding conditions of the i²TransHealth intervention. Engagement with these aspects varied across focus groups, with some topics shared between groups and others unique to specific roles. Among the topics discussed, some perspectives aligned both across and within focus groups, while others diverged, highlighting both shared and contrasting views on what facilitated or hindered the intervention. To reflect these dynamics, the findings are presented thematically, according to the main categories and subcategories, incorporating the range of viewpoints expressed within and between the focus groups.

**Table 1:** Identified main categories and subcategories of the i²TransHealth qualitative process evaluation

**Care of transgender and gender diverse people**
(1) Contact and relationship building (2) Reducing discrimination in care settings (3) Quality of care relationships (4) Heterogeneity and diversity of TGD people seeking care (5) Health burden of TGD people
**Infrastructure and healthcare reality**
(1) Access routes (2) Healthcare gaps (3) Rurality (4) Gatekeeping (5) Social environment of TGD people
**Professional function and professionalisation**
(1) Motivation for healthcare provision and further training (2) Adaptive practice (3) Sense of justice
**Video consultation**
(1) Communication format and setting (2) Practicability for different patient groups (3) Framework conditions (4) Technical problems
**E-health platform**
(1) i²TransHealth website (2) Demand and intensity of use (3) Information and communication features (4) Operational and technical functions
**Networking within i²TransHealth**
(1) Project launch and kick-off event (2) Online round tables (3) Communication between study therapists, study coordination team at UKE, and network physicians

Note. TGD = transgender and gender diverse; UKE = University Medical Centre Hamburg-Eppendorf

#### Care of transgender and gender diverse people

The implementation of i²TransHealth was shaped by processes central to TGD-informed healthcare, as reflected in the focus group data. These included building contacts and relationships, reducing discrimination, improving the quality of care interactions, recognising the diversity of TGD people, and addressing their health burdens.

##### Contact and relationship building:

Across all focus groups, it was evident that TGD people were able to establish contact and build relationships with network physicians and study therapists for general medical, psychiatric, or psychotherapeutic concerns. The network physicians noted the lack of TGD-informed specialists in their respective regions and reported that the i²TransHealth intervention enabled them to make effective referrals for TGD individuals. One study therapist summarised the e-health intervention as a pioneering effort:

*This is perhaps a situation that we may not have in Hamburg* [Note: With a population of nearly 1,900,000 it is the second largest city in Germany] *because there are spaces where people can go and such and they just don’t exist there and then maybe we really ARE somehow one of the first spaces where/ or one of the few spaces where people can turn to.* (T1, study therapist)

Over time, the network physicians observed a decline in direct contact with TGD people receiving online treatment from the study therapists. However, they also reported several new enquiries from TGD people not enrolled in the e-health intervention. The network physicians were seen not exclusively as gateways to the e-health intervention, but also as independent points of contact.

##### Reducing discrimination in care settings:

Recognising the impact of the treatment setting, the network physicians acknowledged the need to reduce discrimination and compensate for systemic inequalities. As a result of the i²TransHealth training, they noted the importance of using the correct form of address and pronouns when registering TGD people. However, they observed that misgendering can unintentionally occur in the busy routines of daily practice, often as a result of discrepancies between a patient’s name and gender presentation and the information recorded on their health insurance card. Some network physicians reported that they had to schedule extra time to offer TGD people a treatment appointment as quickly as possible, while others expressed concern about the additional burdens caused by the COVID-19 pandemic, particularly regarding staffing and administration.

##### Quality of care relationships:

According to the network physicians, many TGD people seeking care were grateful to be treated with humanity and respect. However, some network physicians expressed concern about not always being able to meet the perceived expectations or urgency expressed by some TGD people. The study therapists observed that fully appreciating, understanding, and supporting service users in remote regions was essential for providing effective care. The service users echoed this sentiment and highlighted it through personal expressions of gratitude and satisfaction with both the study therapists and the i²TransHealth intervention overall. They particularly valued the flexibility in scheduling and location of treatment appointments, the openness regarding treatment outcomes, and the helpfulness and empathy of the study therapists in all matters. The following reflection from a service user illustrates how meaningful this openness and support felt in practice:

*And that was super crucial for me*,* to notice from the beginning that that’s okay*,* that an openness to outcome is also desirable and I also have doubts and*,* like*,* inner conflicts somehow/ that I basically knew that I could speak about these things… Exactly*,* yes*,* I found that very*,* very helpful.* (S3, service user)

##### Heterogeneity and diversity of TGD people seeking care:

The network physicians and study therapists perceived TGD people seeking care as a heterogeneous group (e.g., in terms of gender identities or needs for gender-affirming medical treatments). The network physicians, in particular, found working with TGD people enlightening, as their own previous assumptions about universal treatment goals were challenged. They came to recognise the diversity of individual treatment needs. This diversity was also reflected in the ways service users engaged with i²TransHealth. They articulated different healthcare needs and made use of the network of physicians according to their individual requirements – or not at all.

##### Health burden of TGD people:

Possible health burdens of TGD people in general, and service users in particular, were discussed. The network physicians found it particularly difficult to differentiate whether, and which, mental or physical health issues they should address. They encountered TGD people with co-occurring physical conditions, those experiencing psychological distress due to transphobic bullying, and others without additional health concerns. The study therapists also noted that TGD people with multiple health problems and no further connection to the healthcare system can be challenging to accompany. Regardless of this, all service users were generally in better health after the intervention, according to the study therapists. The participating service users reported that they had less self-doubt, less pressure and agonising questions, and a greater sense of wellbeing after the intervention.

#### Infrastructure and healthcare reality

As an e-health intervention developed in response to healthcare shortages, i²TransHealth was affected by various contextual aspects related to infrastructure and the realities of healthcare delivery in remote areas.

##### Access routes:

All focus groups highlighted the reduction or elimination of travel distances for TGD people from remote regions seeking care as a result of the i²TransHealth intervention. At the same time, participants from all focus groups critically noted that travel distances were not completely eliminated for all service users and that costs can arise for face-to-face appointments in addition to video consultations.

##### Healthcare gaps:

In the absence of adequate TGD-informed HCPs, the treating network physicians and study therapists found it difficult to issue referrals in their catchment areas when necessary for a service user. In this regard, one psychiatrist reflected on the isolation some patients face:

*It must be really difficult when you stay in your own city and just can’t find anyone there to talk to*,* no point of contact. (P6*,* psychiatrist)*

Meanwhile, a study therapist highlighted the barriers to collaborative care in more remote regions:

*Co-treatment is basically not possible at all in the most remote areas. (T2*,* study therapist)*

Given the lack of referral options and limited co-treatment opportunities, i²TransHealth emerged as a partial solution. On a positive note, the study therapists emphasised that its low-threshold nature helped them to reach TGD individuals who might otherwise have remained disconnected from healthcare entirely.

##### Rurality:

The network physicians and study therapists linked the missing or infrequent contact with healthcare to the general gaps in care, especially in more rural areas. From the study therapists’ perspective, a lot of educational work had to be done with TGD people from remote regions due to a lack of – or inaccurate – information about gender-affirming care in Germany. In addition, service users described having to cope with the perception that being TGD in predominantly rural communities is often met with limited awareness or ignorance, provoking reactions from confusion to rejection.

##### Gatekeeping:

The study therapists reflected on their own gatekeeper role. They shared the perception that i²TransHealth could be particularly beneficial for service users seeking gender-affirming medical treatments (e.g., hormone therapy) without the need for concurrent mental health support, as these individuals might otherwise have faced years of waiting before receiving any care at all. At the same time, there was a recognition of the limited possibilities:

*I think a lot of things will stick with me*,* but also the perhaps somewhat disillusioning realisation that a model within a system is still within the system. … We still simply have too few therapists*,* too few specialised endocrinologists*,* so all these limits of the health system are still there*,* and we can make a small contribution to making it a little less stupid.* (T3, study therapist)

##### Social environment of TGD people:

A particular concern for the study therapists regarding TGD people from more rural regions was their social environment. This was supported by anecdotal reports from service users about specific experiences of bullying and violence. The study therapists mentioned that they had to psychotherapeutically address the impact of family history, gender role expectations, conservative social environments, bullying, exclusion, and attacks by far-right groups. They also observed that the farther one is from urban centres, the more intensely these dynamics are experienced:

*So far*,* I’ve had the impression that when they talk about their families*,* gender role expectations are very pronounced*,* a girl is like this*,* a boy is like that*,* and that’s how you have to be and nothing else. And also*,* certain currents of political sentiment that I don’t hear very often in the city*,* like certain party names or anecdotes that go in a more than conservative direction. That surprised me – perhaps a bit naively. Well*,* although it’s somehow*,* well*,* clear that those things will emerge*,* but how strongly it seems to coincide with distance from urban areas and how just how STRESSING it is and how very formative it is for the family relationships.* (T5, study therapist)

#### Professional function and professionalisation

A key process within i²TransHealth was the gradual development of TGD-informed expertise, from initial recognition of professional roles to growing professional engagement. This process was particularly evident in the experiences of the network physicians and was also explored by the study therapists, who focused on the ethical dimensions of their professional role.

##### Motivation for healthcare provision and further training:

Several network physicians expressed their interest in and satisfaction with being involved in TGD-informed healthcare and were motivated to participate in additional training to improve the quality of their health services. Their motivation was driven, for example, by witnessing the impact of respectful, inclusive care. As one GP reflected:

*The feedback you get from patients is*,* in some cases*,* really shocking – what they experience at a regular GP practice … I mean*,* I haven’t done much*,* I just listened to this* [Note: i²TransHealth training] *for two days and then said: ‘Okay*,* you can come here.’ But what that alone already meant for the patients – simply being accepted and not being turned away or given a weird look by the medical assistant*,* or being dismissed with ‘I don’t know anything about this*,* go away’ … I feel comfortable being part of this and simply having learned about it and being able to deal with it openly.* (G4, general practitioner)

##### Adaptive practice:

The network physicians were able to establish a stronger connection to the TGD community and their treatment concerns. At the same time, their treatment approach to TGD people varied depending on their practice context, particularly in terms of time, resources, and willingness to adapt. Despite differing levels of adaptation, TGD people were welcomed as a new patient group in many practices involved in the network.

The prior experience and points of contact with TGD people were still partly limited, so the network physicians had to reflect on their professional role and acknowledge their remaining knowledge and competence gaps transparently. The first treatment appointment was crucial in communicating the scope of care available within each practice, facilitating a dialogue that balanced realistic possibilities with valid patient expectations. Some network physicians initially found it difficult to address more private topics such as sexuality and relationships. Over time, they observed a greater awareness and a more natural, confident way of interacting with TGD patients. Working with this population was not perceived as a burden, but as an enrichment.

##### Sense of justice:

Both network physicians and study therapists demonstrated a commitment to supporting TGD people. Many network physicians expressed solidarity with their TGD patients and a desire to counter perceived injustices through dedicated care. One GP shared:

*They always trigger my helper syndrome*,* I notice that. What I notice is that I have this feeling of injustice*,* of a high level of suffering that no one is responsible for. And also the sense that there are still so few points of contact*,* and then I feel the need to take on more than I might normally do. So*,* I have to pay a bit more attention to setting my own boundaries in that regard.* (G2, general practitioner)

Similarly, the study therapists felt that helping TGD people who would otherwise have no point of contact was important work, both personally and politically. They described the moral dilemma of knowing that if a service user requested further care (e.g., outpatient psychotherapy) and no referral options were available, ending the support would amount to rejection, as the service user would be left without care.

#### Video consultation

Video consultations were one of the core components of i²TransHealth. This mode of interaction was discussed exclusively by study therapists and service users, as they were the ones who directly engaged with it. Their experiences with video consultations reflected the structure of the intervention, which involved four months of exclusively online interactions.

##### Communication format and setting:

Among the study therapists, the differences between video consultations and in-person care were emphasised. For some, the lack of face-to-face contact and the limited perception of non-verbal communication and the patient’s body posed a challenge. The online setting was met with ambivalence. Insights into the patient’s home were perceived as helpful and emotionally engaging, but also as something that could blur professional boundaries. These mixed impressions are reflected in the following quotes:

*To see: Where does the person live? What kind of objects are in the room? What kind of room did the person choose for the session? Those are all interesting clues (laughing) for the therapeutic relationship… (T3*,* study therapist)*

*… I do believe that it makes a difference. Whether I’m on the screen*,* but sitting on a patient’s bed*,* so to speak*,* or whether I’m sitting in the UKE consultation room*,* in a professional setting. I sometimes find that this improves the relationship. (T2*,* study therapist)*

Despite these concerns, it was noted that a therapeutic relationship could generally be built in the online format. Service users were overall positive to enthusiastic about the video format, perceiving only minor or occasional differences compared to in-person appointments. One service user, however, highlighted the reduction in non-verbal communication and stressed the need for the initial appointment to take place in person.

##### Practicability for different patient groups:

The study therapists reflected on the suitability and limitations of video-based care. They felt that video consultations worked well for patients who were organised and actively engaged in economic and social life. For less organised patients, quarterly in-person sessions were deemed appropriate. However, patients with personality disorders, a broad spectrum of mental health issues, severe psychological distress, or acute suicidality were regarded as particularly challenging to assess and treat remotely.

##### Framework conditions:

Among the study therapists, maintaining a safe environment and adhering to the video setting was seen as reliant on a fixed schedule of sessions every two weeks. Regarding their working conditions, the study therapists found that face-to-face appointments after the intervention phase helped alleviate pressure by breaking up the sequence of video sessions. The service users also valued the video consultations every two weeks, particularly appreciating the ability to receive care from the comfort of their homes. However, depending on their living situation, they sometimes had to take precautions to ensure privacy. One service user explained:

*And if I’m in my shared flat*,* then I have to think: where do I do it and when do I do it? Or*,* how do I do it so that I can speak freely and don’t somehow have it in the back of my mind that a person is about to come through the door or something. (S2*,* service user)*

##### Technical problems:

The study therapists reported that, occasionally, technical problems disrupted the video consultation (e.g., decoupling of image and sound, no sound, slow or intermittent internet connection). A lack of technical experience among service users, software complexity, and time pressure could further complicate matters. The study therapists discussed it as helpful to be prepared for technical problems and to plan time accordingly. The service users reported no technical issues, except for one who mentioned a problem with internet coverage.

#### E-health platform

The e-health platform was developed as a supporting element of the i²TransHealth intervention. In the focus groups, participants discussed the platform’s public interface, which was used for information and outreach. They also addressed the protected interface, which allowed network physicians to engage with the study team via online forums and enabled service users to communicate with study therapists through a chat function.

##### i²TransHealth website:

Overall, the public interface of the e-health platform, i.e., the i²TransHealth website, was seen as appealing. In terms of outreach efforts, some network physicians noted that the i²TransHealth website provided TGD people with relevant information and facilitated access to network practices. The study therapists observed that service users needed occasional reminders that the website remained a useful resource throughout the e-health intervention. While most service users did not comment on the website, one offered general praise.

##### Demand and intensity of use:

The network physicians reported infrequent use of the protected interface on the e-health platform and were divided on its potential benefits, questioning its necessity and effectiveness. The study therapists observed that service users engaged with the platform to varying degrees, particularly with the chat function. This observation was corroborated by service users themselves, who reported usage ranging from frequent to rare. Low usage was attributed to initial technical problems.

##### Information and communication features:

When discussing platform features, the study therapists specifically mentioned the chat function. They said it reassured them because it made them more accessible to service users. However, they also found 1:1 chat conversations stressful due to their ad hoc and time-consuming nature, as well as the added administrative burden. One study therapist expressed this tension as follows:

*So*,* I want to underline that I somehow find this chat function exhausting*,* because of course: This is a therapeutic contact that takes time. I have to think: What am I going to answer? How deep do I answer/ So*,* no? So*,* at what level do I answer and so on. You can’t do that in passing. … And at the same time*,* I experience a sense of security because of it: That they CAN write to me if something is going on. And easier than by e-mail and I think that’s important because of the physical distance. (T1*,* study therapist)*

In addition, the study therapists held differing views on whether chat conversations encouraged some service users to check in too frequently, exceeding common communication patterns, or if such informal exchanges were generally unproblematic. Meanwhile, the service users concurred that contact via chat was low-threshold and helpful. They used it to clarify questions and difficulties, and it also served as a stabilising factor during times of crisis. One service user described it as helpful and reassuring:

*Well*,* a contact person was there immediately and through the months of working together there was already a relationship of trust*,* that somehow in this psychologically very stressful situation there is a contact person who is directly accessible. I found that TOTALLY helpful*,* and it definitely gave me a sense of security*,* yes. So I was*,* yes*,* very*,* very happy with it. (S3*,* service user)*

In terms of content, both study therapists and service users recommended adding treatment-related FAQs to the platform (e.g., information on health insurance and court applications). The service users also noted the value of including a list of local TGD organisations and services, already available on the public i²TransHealth website. In addition, they suggested that videos featuring TGD-sensitive body exercises would be meaningful and relevant content.

##### Operational and technical functions:

The study therapists noted that the platform could be enhanced in terms of usability and functionality. For instance, certain aspects of their workflows and interactions with service users were hampered. While some service users found the platform to be straightforward, easily accessible, and private, others described it as cumbersome. There were also complaints that service users could not update their personal data. In addition, some service users and network physicians were unaware that they could set up email notifications.

#### Networking within i²TransHealth

Networking within i²TransHealth played an important role as a supporting process of the intervention. In their discussions, the network physicians and study therapists referred to the initial two-day kick-off event, the regular online roundtables, and the continuous communication about patient care.

##### Project launch and kick-off event:

Among the network physicians, the kick-off event was appreciated for providing information about TGD-informed healthcare and networking opportunities. This is reflected in the following statements:

*I benefited greatly from the introductory event. Truly*,* there I received a very good overview of definitions and possibilities*,* operative and pharmaceutical interventions – that was really positive. (P4*,* psychiatrist)*

*It was nice to just meet colleagues*,* so we even had psychiatric colleagues from the local area here who I had never seen before*,* only briefly communicated with*,* and that was EXTREMELY important. (G1*,* general practitioner)*

The kick-off event was seen as important preparation for delivering appropriate healthcare to TGD patients, or as a means of ensuring it. This view was supported by a network psychiatrist, who reported that missing the event made it difficult to get started.

##### Online roundtables:

The network physicians were also pleased with the optional online roundtables for keeping them informed, facilitating their work, and fostering a sense of professional community. There was little regret about not meeting in person. Online participation was deemed more feasible than in-person appointments, but some were unable to join due to workload constraints. Among the study therapists, there was the perception that some network physicians did not participate at all; at the same time, many interested network physicians were noted.

##### Communication between study therapists, study coordination team at UKE, and network physicians:

Several network physicians stated that the study therapists and study coordination team were qualified and reliable contacts. However, the network physicians and study therapists agreed that information about patient referrals was not sufficiently organised. While the network physicians wished to be more involved, the study therapists raised the issues related to time constraints, confidentiality obligations, and the need to clarify processes and responsibilities. One study therapist summarised the situation as follows:

*Yes*,* and I also know that some of the network partners were a bit frustrated that the communication didn’t always work out so well*,* they would have liked to be more involved in the processes*,* but there really wasn’t any time for that*,* to update them on who has landed where and is at what stage and so on. (T3*,* study therapist)*

## Discussion

The results of the qualitative process evaluation provide important insights into both the implementation of this specific intervention and transferable lessons for e-health models tailored to TGD care more broadly. Drawing on the experiences of the service users, network physicians, and study therapists of the i²TransHealth intervention, we derive recommendations to digitally enhance, expand, and sustain care provision in underserved regions, as well as to inform the development and design of similar interventions in other contexts. In the following discussion, we build on the specific findings from the implementation of i²TransHealth (including the evaluation of key e-health components such as video consultations and chat-based support) to formulate overarching recommendations for the design and delivery of TGD-informed e-health interventions. These insights aim to guide both the future implementation of i²TransHealth and the development of new, context-sensitive digital care models for TGD populations. The findings indicate that the video consultations, along with local GP and psychiatric care, were successfully implemented.

However, while the study therapists were able to tailor their support to individual service users, their efforts were constrained by the primarily video- and chat-based nature of the intervention during the RCT period and the shortage of TGD-informed HCPs in remote areas. A more flexible approach, combining in-person care with video consultations at all times, could be a feasible expansion in the future. Furthermore, it seems essential to expand the physician network and to provide financial backing for TGD-sensitive training across regional care structures. Another important result is that the network physicians not only facilitated access to the e-health intervention and medical support for service users in case of need, but also provided healthcare for TGD people who were unable or unwilling to participate in the study (e.g., due to age). Moving forward, collaboration should focus on coordinating interdisciplinary, specialised TGD-informed healthcare in metropolitan regions, and GP and psychiatric care in remote areas for TGD people. The study’s core framework – implementing an e-health intervention, fostering cross-sectoral networking, and advancing HCP training as interconnected elements – has proven viable and should be further refined.

The following sections provide specific recommendations (cf. Table 2) for enhancing and implementing cross-sectoral e-health care for TGD people in remote regions, drawing on the study findings:

**Table 2:** Key recommendations for enhancing cross-sectoral e-health care for TGD people in remote regions

**Setting up care for TGD people from remote areas**	Expand TGD-informed HCP training, create a public directory of competent providers, develop tailored care concepts based on health burden, and embed e-health effectively
**Dealing with deficiencies in infrastructure and healthcare reality**	Enhance e-health flexibility, offer financial support for TGD service users, and increase government investment in health and digital infrastructure in underserved regions
**Promoting professional function and professionalisation**	Provide formal training on referrals and administrative processes, establish clear practice guidelines per provider group, ensure continuous supervision, and certify TGD-informed training with visible accreditation
**Customising video consultations**	Combine e-health and in-person care, adjust care frameworks dynamically, implement offline tasks to prevent screen fatigue, and offer patient training (e.g., guides and video tutorials) for technical skills
**Outreach**,** information**,** and communication via e-health platform**	Expand FAQs, create a dedicated platform for service users and therapists, allocate sufficient chat consultation time, enable availability notifications, and continuously improve technical functionality
**Strengthen networking**	Facilitate referral feedback while ensuring data protection and fostering patient-centred collaboration

Note. FAQs = frequently asked questions; TGD = transgender and gender diverse; HCP = healthcare professional

### Setting up care for transgender and gender diverse people from remote areas

The main goal of facilitating initial access to TGD-informed healthcare across distance – both remotely via e-health and in person through the network of physicians – was achieved. The appreciation, understanding, and expertise of the study therapists and network physicians were beneficial, which ties in with the high demand for culturally competent and diversity-sensitive trained HCPs [8, 32–36]. The service users appreciated the individualised approach to care concerns in line with current TGD health guidelines [37–40]. However, ongoing education and reflection are needed to ensure more TGD-informed HCPs for TGD people nationwide and to avoid discrimination in healthcare (e.g., cis-normative assumptions, unintended or insensitive language) [11, 32, 35, 41–44]. Clarifying co-occurring health issues remains a challenge, which is why face-to-face initial and follow-up consultations play a crucial role in complementing video consultations [1, 45]. It should not be forgotten that, for many TGD people, including our service users, mental health support is tied to obtaining a possible referral letter for gender-affirming medical treatments [39, 40, 46]. Therefore, in the long run, linking e-health and outpatient care services for TGD people who want to manage parts of their medical transition independently of their place of residence is to be favoured [1, 4, 47–49]. From our findings, we derive the following recommendations: expansion of TGD-informed healthcare training for HCPs, a publicly accessible directory of culturally competent and diversity-sensitive HCPs, tailor-made concepts for TGD people adapted to health burden level, and meaningful embedding of e-health.

### Dealing with deficiencies in infrastructure and healthcare reality

In fact, the video consultations reduced travel distances and costs, thereby helping to mitigate a barrier to TGD-informed healthcare [1, 4, 49]. Connecting service users to i²TransHealth made it possible to address healthcare gaps locally, but an e-health intervention did not replace missing infrastructure. Referral, if needed, was extremely difficult in the catchment area of the service users. However, TGD people from remote areas could be reached through i²TransHealth and were no longer isolated (especially in more rural areas), contributing to improved accessibility, education, and participation in TGD-informed healthcare [2, 34]. TGD people seeking care could be served more flexibly by means of e-health services, independent of their place of residence, in order to ease the gatekeeping problem in TGD-informed healthcare [42, 50, 51]. Regardless of our study, the lack of digitalisation in very remote regions remains a problem as long as local decision-makers do not invest in infrastructure [52–57]. In our view, this leads to the following recommendations: enable flexibility through e-health for care seekers and care providers, offer compensation and reimbursement options for care seeking TGD people from remote regions to address regional care deficits, and government investment in underserved regions in health and digital infrastructure.

### Promoting professional function and professionalisation

Our results suggest that HCPs first need to become aware of their professional function in TGD-informed healthcare and recognise the need for further professionalisation (e.g., additional training) through initial trainings such as those provided by i²TransHealth (i.e., in our case kick-off event, online round tables, contact via messages and phone). Our findings confirm that for a development process from an inexperienced to a TGD-informed HCP, expert support is imperative [8, 11, 32, 35, 43]. Expressions of solidarity by HCPs for TGD people do not outweigh the fact that injustices within their own sphere of influence in healthcare can only be reduced piece by piece [3, 8, 11, 42, 58–62]. On the other hand, raising awareness of TGD-informed healthcare can be sustainable once it has taken place. More resources and willingness to change could be considered by many HCPs in the longer term [35, 42]. Future TGD health services need to expand support opportunities for inexperienced HCPs so that professionalisation in TGD-informed healthcare is integrated into professional self-understanding [11, 63–65]. Based on our findings, we recommend: formal training on administrative aspects of TGD-informed healthcare (e.g., referrals, communication with institutions like health insurance providers or registry offices), practice guidelines tailored to each care provider group with clear delineation of responsibilities, continuous supervision and intervision to ensure awareness and competence of HCPs, and certification of TGD-informed training, visibly displayed in practices and on websites.

### Customising video consultations

Our findings are consistent with research indicating different perspectives on mental e-health between HCPs and patients [66–72]. From the perspective of study therapists, the reduction of non-verbal and physical cues, as well as formal requirements, proved to be relevant, whereas service users found the video format more accessible and easier to engage with. While research indicates that e-health interventions can be helpful even for severely burdened patients [73], our findings provide more nuanced information about which severely burdened TGD people need additional support (i.e., TGD people with personality disorders, a broad spectrum of mental health issues, a high level of psychological distress, or suicide risk). In addition, the framework conditions of e-health and technical issues need to be considered [74–79]. The following recommendations can be derived from the results: regular face-to-face appointments in addition to video consultations, negotiation and adaptation of the framework conditions at the beginning and during care, regular offline tasks for therapists to prevent screen fatigue, and optional patient training (e.g., written instructions plus video tutorial) to increase the technical skills of service users.

### Outreach, information and communication via e-health platform

The results indicate that the i²TransHealth website performed well in providing information and access to TGD-informed healthcare. However, a meaningful addition would be a set of FAQs on formal procedures that regularly arise in the care of TGD people. Regarding the protected interface of the e-health platform, the results are mixed. The low uptake by the network physicians is probably due to its novelty and the ease with which it could be bypassed in favour of established, traditional communication channels such as phone and email. In contrast, the service users seemed to be using the platform as needed. The chat conversations provided a low-threshold contact between the service users and the study therapists, but also resulted in increased workload and stress for the study therapists. They should therefore be supported in order to minimise the administrative burden and manage expectations of availability [70, 72]. Furthermore, technical issues hampered their work at times. We therefore recommend the following: expansion of the existing FAQs, a dedicated platform exclusively for service users and study therapists (as network physicians preferred traditional communication channels), sufficient time allocation for chat conversations, the option to notify service users of unavailability via chat in cases of high workload, and continuous monitoring and improvement of technical functions and platform operation.

### Strengthen networking

The kick-off event and online roundtables effectively facilitated networking and equipped network physicians to provide healthcare for TGD people. In addition to offering knowledge on TGD-informed healthcare and ongoing support, the following recommendations can further improve interdisciplinary networking: providing feedback on patient referrals while ensuring compliance with data protection regulations and fostering patient-centred collaboration where appropriate.

### Strengths and limitations

A key strength of the study is that it includes multiple stakeholder perspectives – network physicians, study therapists, and service users – offering a rich account of i^2^TransHealth’s implementation. However, the relatively small number of participants limited the extent to which data saturation could be achieved, particularly among network physicians and study therapists [80]. Furthermore, the complex interactions that characterise focus groups [81, 82] often make it neither feasible nor meaningful to quantify opinions in a definitive manner. Consequently, only general trends can be depicted, and the significance of individual statements must be carefully assessed.

Another limitation is that the participant dynamics in the focus groups with the network physicians and study therapists differed from those of the service users. In focus groups, participants often deepen or broaden statements and viewpoints due to group dynamics. Notably, while the focus groups with network physicians and study therapists were rich and dynamic, the service users engaged less with each other. This may be because they were heterogeneously sampled and unfamiliar with one another, making it difficult to establish rapport and to find common ground.

Finally, it should be noted that the focus groups were conducted by the study coordination team (AD, TN) and the scientific staff of i²TransHealth (JR), which may have influenced participants’ openness. Despite this, critical issues were raised in all focus groups, indicating the quality of the data. To further ensure a balanced interpretation and reduce potential bias, the data were analysed collaboratively by a study team member (JR) and a researcher external to the study (FS). The data were collected online and were of good quality overall. Nevertheless, a few text segments that seemed interesting could not, or could not completely, be analysed due to temporary technical problems.

### Implications for future research

The i²TransHealth infrastructure holds high transfer potential for broader healthcare applications, specifically within TGD-informed healthcare and the dissemination of university-based specialised medicine. Our findings underscore the necessity of expanding e-health services and fostering specialised training and networking among HCPs to enhance outpatient care. In the medium term, this could enable TGD people to find suitable HCPs in their immediate vicinity for their healthcare needs. As a pioneering e-health study, i²TransHealth could only evaluate a limited scope of TGD-informed health services. In the long term, the comprehensive integration of online and offline health services across all aspects of TGD-informed healthcare should be evaluated in a longitudinal study. Even today, a number of treatment appointments can be made online with the appropriate equipment, which could contribute to significant time and cost savings for service users [57, 83, 84]. Expanding online training for HCPs is crucial [85], yet further in-depth research is needed to determine the optimal training format. Both asynchronous e-learning modules and interactive online group seminars warrant rigorous investigation [86–92]. The ongoing digitalisation of healthcare offers significant benefits for both care seekers and care providers [17, 93], but careful methodological guidance is necessary, as e-health in TGD-informed healthcare is still in its early stages [12, 28].

## Conclusions

The qualitative process evaluation of i²TransHealth underscores significant deficits in TGD-informed healthcare, especially in remote areas. TGD people appreciate the flexibility of e-health services, especially video consultations with affirming, knowledgeable therapists. However, these remote sessions posed challenges for therapists when users were highly distressed, highlighting the need for swift referral pathways for acute care. Expanding the number of TGD-informed HCPs, such as our network physicians, is essential. Moreover, sustainable e-health services require institutional and policy support. While digitalisation, specialised HCP training, and individualised care options present societal challenges beyond e-health alone, the substantial benefits for service users – who finally gain access to TGD-informed healthcare – must be emphasised. Video- and chat-based formats are becoming increasingly relevant and should be flexibly tailored to meet users’ levels of distress and digital needs, ensuring inclusive and effective care for the TGD population.

## Supplementary Information

Below is the link to the electronic supplementary material.

Supplementary Material 1

## Data Availability

The datasets generated and/or analysed during the current study are not publicly available, but may be available from the corresponding author on a reasonable request. The quotations and the full interview guide are available in their original German version and can be provided only upon justified request.

## Footnotes

1 One practice manager from one of the psychiatric practices also participated in this focus group. For simplicity, all participants in this group are hereafter referred to as ‘psychiatrists’.

## Abbreviations

FAQs: Frequently asked questions
GPs: General practitioners
HCPs: Healthcare professionals
i²TransHealth: Interdisciplinary, internet-based transgender and gender diverse healthcare intervention
ICT: Information and communication technologies
RCT: Randomised controlled trial
TGD: Transgender and gender diverse
UKE: University Medical Centre Hamburg-Eppendorf

## References

[1] Ross MB, Van De Grift TC, Elaut E, Nieder TO, Becker-Hebly I, Heylens G, et al. Experienced barriers of care within European treatment seeking transgender individuals: A multicenter ENIGI follow-up study. Int J Transgender Heal. 2023;24(1):26–37. 10.1080/26895269.2021.1964409 PMC9879197 36713146

[2] Renner J, Blaszcyk W, Täuber L, Dekker A, Briken P, Nieder TO. Barriers to accessing health care in rural regions by Transgender, Non-Binary, and gender diverse people: A Case-Based scoping review. Front Endocrinol (Lausanne). 2021;12:717821. 34867775 10.3389/fendo.2021.717821 PMC8637736

[3] Whitehead J, Shaver J, Stephenson R. Outness, Stigma, and primary health care utilization among rural LGBT populations. PLoS ONE. 2016;11(1):e0146139. 26731405 10.1371/journal.pone.0146139 PMC4701471

[4] Koehler A, Strauss B, Briken P, Szuecs D, Nieder TO. Centralized and decentralized delivery of transgender health care services: A systematic review and a global expert survey in 39 countries. Front Endocrinol (Lausanne). 2021;12:717914. 34630327 10.3389/fendo.2021.717914 PMC8497761

[5] Castro Varela M. do M. “… nicht so greifbar und doch real”. Eine quantitative und qualitative Studie zu Gewalt- und (Mehrfach-) Diskriminierungserfahrungen von lesbischen, bisexuellen Frauen und Trans* in Deutschland. [Internet]. LesMigraS; 2012. Available from: https://lesmigras.de/wp-content/uploads/2021/11/Dokumentation-Studie-web_sicher.pdf.

[6] European Union Agency for Fundamental Rights. LGBTI II: a long way to go for LGBTI equality [Internet]. Luxembourg. 2020. Available from: https://fra.europa.eu/sites/default/files/fra_uploads/fra-2020-lgbti-equality-1_en.pdf.

[7] European Union Agency for Fundamental Rights. LGBTIQ at a crossroads: progress and challenges [Internet]. Luxembourg. 2024. Available from: https://fra.europa.eu/sites/default/files/fra_uploads/fra-2024-lgbtiq-equality_en.pdf.

[8] Kano M, Silva-Bañuelos AR, Sturm R, Willging CE. Stakeholders’ recommendations to improve Patient-centered LGBTQ primary care in rural and multicultural practices. J Am Board Fam Med JABFM. 2016;29(1):156–60. 26769889 10.3122/jabfm.2016.01.150205

[9] Eliason MJ, Hughes T. Treatment counselor’s attitudes about lesbian, gay, bisexual, and transgendered clients: urban vs. rural settings. Subst Use Misuse. 2004;39(4):625–44. 15115216 10.1081/ja-120030063

[10] Logie CH, Lys CL, Dias L, Schott N, Zouboules MR, MacNeill N, et al. Automatic assumption of your gender, sexuality and sexual practices is also discrimination: exploring sexual healthcare experiences and recommendations among sexually and gender diverse persons in Arctic Canada. Health Soc Care Community. 2019;27(5):1204–13. 30989767 10.1111/hsc.12757

[11] Sharma A, Shaver JC, Stephenson RB. Rural primary care providers’ attitudes towards sexual and gender minorities in a Midwestern state in the USA. Rural Remote Health. 2019;19(4):5476. 31675243 10.22605/RRH5476

[12] Renner J, Täuber L, Nieder TO. Need for inclusive consideration of transgender and gender diverse people in E-Health services: A systematic review. J Clin Med. 2022;11(4):1090. 35207359 10.3390/jcm11041090 PMC8880545

[13] Asaad M, Rajesh A, Vyas K, Morrison SD. Telemedicine in transgender care: A Twenty-First-Century beckoning. Plast Reconstr Surg. 2020;146:E108–9. 10.1097/PRS.0000000000006935 32590687

[14] Hamnvik O-PR, Agarwal S, AhnAllen CG, Goldman AL, Reisner SL. Telemedicine and inequities in health care access: the example of transgender health. Transgender Heal. 2020;7(2):113–6. 10.1089/trgh.2020.0122 PMC9829126 36644516

[15] Stewart MK, Allison MK, Hunthrop MSG, Marshall SA, Cornell CE. Outcomes research on Telemedicine-Delivered Gender-Affirming health care for transgender youth is needed now: A call to action. Transgender Heal. 2023;8(1):1–5. 10.1089/trgh.2021.0063 PMC9942169 36824385

[16] Nieder TO, Renner J, Zapf A, Sehner S, Hot A, König H-H, et al. Interdisciplinary, internet-based trans health care (i^2^TransHealth): study protocol for a randomised controlled trial. BMJ Open. 2022;12(2):e045980. 35105559 10.1136/bmjopen-2020-045980 PMC8808412

[17] Andersson G. Internet interventions: Past, present and future. Internet Interv. 2018;12:181–8. 30135782 10.1016/j.invent.2018.03.008 PMC6096319

[18] Andersson G, Cuijpers P. Internet-Based and other computerized psychological treatments for adult depression: A Meta-Analysis. Cogn Behav Ther. 2009;38(4):196–205. 20183695 10.1080/16506070903318960

[19] Königbauer J, Letsch J, Doebler P, Ebert D, Baumeister H. Internet- and mobile-based depression interventions for people with diagnosed depression: A systematic review and meta-analysis. J Affect Disord. 2017;223:28–40. 28715726 10.1016/j.jad.2017.07.021

[20] Sood S, Mbarika V, Jugoo S, Dookhy R, Doarn CR, Prakash N, et al. What is telemedicine? A collection of 104 Peer-Reviewed perspectives and theoretical underpinnings. Telemed e-health. 2007;13(5):573–90. 10.1089/tmj.2006.0073 17999619

[21] Erbe D, Eichert H-C, Riper H, Ebert DD. Blending Face-to-Face and Internet-Based interventions for the treatment of mental disorders in adults: systematic review. J Med Internet Res. 2017;19(9):e6588. 10.2196/jmir.6588 PMC5622288 28916506

[22] Fraser L, Etherapy. Ethical and clinical considerations for version 7 of the world professional association for transgender health’s standards of care. Int J Transgenderism. 2009;11(4):247–63.

[23] Radix AE, Bond K, Carneiro PB, Restar A. Transgender individuals and digital health. Curr HIV/AIDS Rep. 2022;19(6):592–9. 36136217 10.1007/s11904-022-00629-7 PMC9493149

[24] Nieder TO, Renner J, Sehner S, Pepić A, Zapf A, Lambert M, et al. Effect of the i^2^TransHealth e-health intervention on psychological distress among transgender and gender diverse adults from remote areas in germany: a randomised controlled trial. Lancet Digit Heal. 2024;6(12):e883–93. 10.1016/S2589-7500(24)00192-4 39419729

[25] Renner J, Briken P, Dekker A, Pregartbauer L, Zapf A, Sehner S, et al. Which transgender and gender diverse groups benefit most from E-health? Subgroup analyses of the randomized controlled trial i^2^TransHealth in Germany. Int J Transgender Heal. 2025. 10.1080/15532739.2025.2465710 PMC13015102 41891088

[26] Grochtdreis T, Konnopka A, Renner J, König H-H, Dekker A, Briken P, et al. Excess costs of transgender and gender-diverse people with gender incongruence and gender dysphoria compared with people from the general population in germany: a secondary analysis using data from a randomised controlled trial and a representative Teleph. BMJ Open. 2025;15(4):e089663. 40204312 10.1136/bmjopen-2024-089663 PMC11987145

[27] Grochtdreis T, König H-H, Renner J, Sehner S, Dekker A, Briken P, et al. Cost-Effectiveness of an Interdisciplinary, Internet-Based transgender health care program in germany: economic evaluation alongside a randomized controlled trial. J Med Internet Res. 2025;27(1):e66371. 40537093 10.2196/66371 PMC12202241

[28] Moore GF, Audrey S, Barker M, Bond L, Bonell C, Hardeman W, et al. Process evaluation of complex interventions: medical research Council guidance. BMJ. 2015;350:h1258. 25791983 10.1136/bmj.h1258 PMC4366184

[29] Helfferich C. Die Qualität qualitativer Daten. Manual für die Durchführung qualitativer interviews. 4th ed. Wiesbaden: VS Verlag für Sozialwissenschaften; 2011.

[30] Kuckartz U, Rädiker S. Qualitative Inhaltsanalyse. Methoden, Praxis, Computerunterstützung. 5th ed. Weinheim, Basel: Beltz Juventa; 2022.

[31] Kuckartz U. Qualitative text Analysis. A guide to Methods, practice & using software. London: SAGE; 2014.

[32] Baiocco R, Pezzella A, Pistella J, Kouta C, Rousou E, Rocamora-Perez P, et al. LGBT + Training needs for health and social care professionals: A Cross-cultural comparison among seven European countries. Sex Res Soc Policy. 2021;2021:1–15.

[33] Hammer MR. The developmental paradigm for intercultural competence research. Int J Intercult Relations. 2015;48:12–3.

[34] McCann E, Sharek D. Mental health needs of people who identify as transgender: A review of the literature. Arch Psychiatr Nurs. 2016;30(2):280–5. 26992883 10.1016/j.apnu.2015.07.003

[35] van Heesewijk J, Kent A, van de Grift TC, Harleman A, Muntinga M. Transgender health content in medical education: a theory-guided systematic review of current training practices and implementation barriers & facilitators. Adv Heal Sci Educ. 2022;27(3):817–46. 10.1007/s10459-022-10112-y PMC9374605 35412095

[36] Pratt-Chapman ML, Eckstrand K, Robinson A, Beach LB, Kamen C, Keuroghlian AS, et al. Developing standards for cultural competency training for health care providers to care for Lesbian, Gay, Bisexual, Transgender, Queer, Intersex, and asexual persons: consensus recommendations from a National panel. LGBT Heal. 2022;9(5):340–7. 10.1089/lgbt.2021.0464 PMC9291720 35443812

[37] Hembree WC, Cohen-Kettenis PT, Gooren L, Hannema SE, Meyer WJ, Murad MH, et al. Endocrine treatment of gender-dysphoric/ gender-incongruent persons: an endocrine society clinical practice guideline. J Clin Endocrinol Metab. 2017;102(11):3869–903. 28945902 10.1210/jc.2017-01658

[38] T’Sjoen G, Arcelus J, De Vries ALC, Fisher AD, Nieder TO, Özer M, et al. European society for sexual medicine position statement assessment and hormonal management in adolescent and adult trans People, with attention for sexual function and satisfaction. J Sex Med. 2020;17(4):570–84. 32111534 10.1016/j.jsxm.2020.01.012

[39] Coleman E, Radix AE, Bouman WP, Brown GR, de Vries ALC, Deutsch MB, et al. Standards of care for the health of transgender and gender diverse People, version 8. Int J Transgender Heal. 2022;23(sup1):S1–259. 10.1080/26895269.2022.2100644 PMC9553112 36238954

[40] Deutsche Gesellschaft für Sexualforschung. S3-Leitlinie zur Diagnostik, Beratung und Behandlung im Kontext von Geschlechtsinkongruenz, Geschlechtsdysphorie und Trans-Gesundheit. AWMF-Registernummer 138-001 [Internet]. 2018. Available from: https://register.awmf.org/de/leitlinien/detail/138-001.

[41] Loo S, Almazan AN, Vedilago V, Stott B, Reisner SL, Keuroghlian AS. Understanding community member and health care professional perspectives on gender-affirming care—A qualitative study. PLoS ONE. 2021;16(8):e0255568. 34398877 10.1371/journal.pone.0255568 PMC8366980

[42] de Vries E, Kathard H, Müller A, Debate. Why should gender-affirming health care be included in health science curricula? BMC Med Educ. 2020;20(1):1–10. 10.1186/s12909-020-1963-6 PMC7023748 32059721

[43] Stroumsa D, Shires DA, Richardson CR, Jaffee KD, Woodford MR. Transphobia rather than education predicts provider knowledge of transgender health care. Med Educ. 2019;53(4):398–407. 30666699 10.1111/medu.13796

[44] Stryker SD, Pallerla H, Yockey RA, Bedard-Thomas J, Pickle S. Training mental health professionals in Gender-Affirming care: A survey of experienced clinicians. Transgender Heal. 2022;7(1):68–77. 10.1089/trgh.2020.0123 PMC9829147 36644027

[45] Bukowski LA, Blosnich J, Shipherd JC, Kauth MR, Brown GR, Gordon AJ. Exploring rural disparities in medical diagnoses among veterans with transgender-related diagnoses utilizing veterans health administration care. Med Care. 2017;55(Number 9 Suppl 2):S97–103. 10.1097/MLR.0000000000000745 28806372

[46] Nieder TO, Strauß B. S3-Leitlinie Zur Diagnostik, beratung und behandlung Im kontext von Geschlechtsinkongruenz, geschlechtsdysphorie und Trans-Gesundheit. Z für Sex. 2019;32(2):70–9.

[47] de Brouwer IJ, Elaut E, Becker-Hebly I, Heylens G, Nieder TO, van de Grift TC, et al. Aftercare needs following gender-affirming surgeries: findings from the ENIGI multicenter European follow-up study. J Sex Med. 2021. 10.1016/j.jsxm.2021.08.005 34548264

[48] Hughto JMW, Murchison GR, Clark K, Pachankis JE, Reisner SL. Geographic and individual differences in healthcare access for U.S. Transgender adults: A multilevel analysis. LGBT Heal. 2016;3(6):424–33. 10.1089/lgbt.2016.0044 PMC5165678 27636030

[49] Eyssel J, Koehler A, Dekker A, Sehner S, Nieder TO. Needs and concerns of transgender individuals regarding interdisciplinary transgender healthcare: A non-clinical online survey. PLoS ONE. 2017;12(8):e0183014. 28846715 10.1371/journal.pone.0183014 PMC5573291

[50] Dewey JM, Gesbeck MM. (Dys) functional diagnosing: mental health Diagnosis, Medicalization, and the making of transgender patients. Humanity Soc. 2017;41(1):37–72.

[51] Smiley A, Burgwal A, Orre C, Summanen E, Nieto IG, Vidić J, et al. Overdiagnosed but underserved. Poland, Serbia, Spain, and Sweden: Trans Health Survey.: Trans Healthcare in Georgia; 2017.

[52] Ex P, Amelung VE. Auf und ab: der politische Wille Zur Stärkung der digitalisierung Im gesundheitswesen. Gesundheits- Und Sozialpolitik. 2018;72(2):26–30.

[53] Flottorp SA, Oxman AD, Krause J, Musila NR, Wensing M, Godycki-Cwirko M, et al. A checklist for identifying determinants of practice: A systematic review and synthesis of frameworks and taxonomies of factors that prevent or enable improvements in healthcare professional practice. Implement Sci. 2013;8(1):1–11. 23522377 10.1186/1748-5908-8-35 PMC3617095

[54] Nohl-Deryk P, Brinkmann JK, Gerlach FM, Schreyögg J, Achelrod D. Barriers to digitalisation of healthcare in germany: A survey of experts. Gesundheitswesen. 2018;80(11):939–45. 29301149 10.1055/s-0043-121010

[55] Ammenwerth E, Duftschmid G, Al-Hamdan Z, Bawadi H, Cheung NT, Cho KH, et al. International comparison of six basic eHealth indicators across 14 countries: an eHealth benchmarking study. Methods Inf Med. 2020;59(2):E46–63. 33207386 10.1055/s-0040-1715796 PMC7728164

[56] Thye J, Hübner U, Hüsers J, Babitsch B. IT decision making in German Hospitals – Do ceos open the black box? Stud Health Technol Inf. 2017;243:112–6. 28883182

[57] Brönneke JB, Debatin JF. Digitalisierung Im gesundheitswesen und Ihre effekte auf die Qualität der gesundheitsversorgung. Bundesgesundheitsblatt - Gesundheitsforsch - Gesundheitsschutz. 2022;65(3):342–7. 10.1007/s00103-022-03493-3 PMC8856867 35181795

[58] Sinnard MT, Raines CR, Budge SL. The association between geographic location and anxiety and depression in transgender individuals: an exploratory study of an online sample. Transgender Heal. 2016;1(1):181–6. 10.1089/trgh.2016.0020 PMC5367480 28861531

[59] Edmiston EK, Donald CA, Sattler AR, Peebles JK, Ehrenfeld JM, Eckstrand KL. Opportunities and gaps in primary care preventative health services for transgender patients: A systemic review. Transgender Heal. 2016;1(1):216–30. 10.1089/trgh.2016.0019 PMC5367473 28861536

[60] Hughto JMW, Reisner SL, Pachankis JE. Transgender stigma and health: A critical review of stigma determinants, mechanisms, and interventions. Soc Sci Med. 2015;147:222–31. 26599625 10.1016/j.socscimed.2015.11.010 PMC4689648

[61] Lykens JE, LeBlanc AJ, Bockting WO. Healthcare experiences among young adults who identify as genderqueer or nonbinary. LGBT Heal. 2018;5(3):191–6. 10.1089/lgbt.2017.0215 PMC6016091 29641314

[62] Ross KAE, Law MP, Bell A. Exploring healthcare experiences of transgender individuals. Transgender Heal. 2016;1(1):238–49. 10.1089/trgh.2016.0021 PMC5367478 28861538

[63] Nieder TO, Güldenring A, Woellert K, Briken P, Mahler L, Mundle G. Ethical aspects of mental health care for Lesbian, Gay, Bi-, Pan-, Asexual, and transgender people: A Case-based approach. Yale J Biol Med. 2020;93(4):593–602. 33005124 PMC7513438

[64] Suess Schwend A. Trans health care from a depathologization and human rights perspective. Public Health Rev. 2020;41(1):3. 32099728 10.1186/s40985-020-0118-y PMC7031999

[65] Nieder TO, Koehler A, Briken P, Eyssel J. Mapping key stakeholders’ position towards interdisciplinary transgender healthcare: A stakeholder analysis. Health Soc Care Community. 2020;28(2):385–95. 31573123 10.1111/hsc.12870

[66] Lopez A, Schwenk S, Schneck CD, Griffin RJ, Mishkind MC. Technology-Based mental health treatment and the impact on the therapeutic alliance. Curr Psychiatry Rep. 2019;21(8):1–7. 31286280 10.1007/s11920-019-1055-7

[67] Legerer-Bratengeyer A. Zoom-Fatigue managen. Psychother Forum. 2021;25(3):109–14.

[68] Shklarski L, Abrams A, Bakst E. Navigating changes in the physical and psychological spaces of psychotherapists during Covid-19: when home becomes the office. Pract Innov. 2021;6(1):55–66.

[69] Hilty DM, Armstrong CM, Smout SA, Crawford A, Maheu MM, Drude KP, et al. Findings and guidelines on provider Technology, Fatigue, and Well-being: scoping review. J Med Internet Res. 2022;24(5):e34451. 35612880 10.2196/34451 PMC9178447

[70] McCord C, Bernhard P, Walsh M, Rosner C, Console K. A consolidated model for telepsychology practice. J Clin Psychol. 2020;76(6):1060–82. 32285940 10.1002/jclp.22954 PMC7383805

[71] Wentzel J, van der Vaart R, Bohlmeijer ET, van Gemert-Pijnen JEWC. Mixing online and Face-to-Face therapy: how to benefit from blended care in mental health care. JMIR Ment Heal. 2016;3(1):e9. 10.2196/mental.4534 PMC4764785 26860537

[72] van Leeuwen H, Sinnaeve R, Witteveen U, Van Daele T, Ossewaarde L, Egger JIM, et al. Reviewing the availability, efficacy and clinical utility of telepsychology in dialectical behavior therapy (Tele-DBT). Borderline Personal Disord Emot Dysregulation. 2021;8(1):1–15. 10.1186/s40479-021-00165-7 PMC8556811 34717772

[73] Weisel KK, Lehr D, Heber E, Zarski A-C, Berking M, Riper H, et al. Severely burdened individuals do not need to be excluded from Internet-Based and Mobile-Based stress management: effect modifiers of treatment outcomes from three randomized controlled trials. J Med Internet Res. 2018;20(6):e9387. 10.2196/jmir.9387 PMC6030574 29921562

[74] Vriends J, Symonds-Brown H. The experiences of transgender and gender diverse children and youth using telehealth: a meta-ethnography. Int J Transgender Heal. 2024;1–21.

[75] Inwards-Breland DJ, Yeh D, Marinkovic M, Richardson TR, Marino-Kibbee B, Bayley A, et al. Facilitators and barriers to using telemedicine for gender-affirming care in gender-diverse youth: a qualitative study. J Telemed Telecare. 2024. 10.1177/1357633X241231015 PMC12179396 38400512

[76] Smalley JM, Lozano JM, McMahon CJ, Colburn JA. Improving global access to transgender health care: outcomes of a telehealth quality improvement study for the air force transgender program. Transgender Heal. 2022;7(2):150–8. 10.1089/trgh.2020.0167 PMC9829134 36644511

[77] Stoehr JR, Jahromi AH, Hunter EL, Schechter LS. Telemedicine for Gender-Affirming medical and surgical care: A systematic review and Call-to-Action. Transgender Heal. 2021;7(2):117–26. 10.1089/trgh.2020.0136 PMC9829135 36644513

[78] Hertling S, Hertling D, Martin D, Graul I. Acceptance, Use, and barriers of telemedicine in transgender health care in times of SARS-CoV-2: nationwide Cross-sectional survey. JMIR Public Heal Surveill. 2021;7(12):e30278. 10.2196/30278 PMC8647970 34591783

[79] Sequeira GM, Kidd KM, Coulter RWS, Miller E, Fortenberry D, Garofalo R, et al. Transgender youths’ perspectives on telehealth for delivery of Gender-Affirming care. J Adolesc Heal. 2021;68(6):1207–10. 10.1016/j.jadohealth.2020.08.028 PMC7510534 32980246

[80] Rahimi S, Khatooni M. Saturation in qualitative research: an evolutionary concept analysis. Int J Nurs Stud Adv. 2024;6:100174. 38746797 10.1016/j.ijnsa.2024.100174 PMC11080421

[81] Wong LP. Focus group discussion: a tool for health and medical research. Singap Med J. 2008;49(3):256–60. 18363011

[82] Sim J, Waterfield J. Focus group methodology: some ethical challenges. Qual Quant. 2019;53(6):3003–22.

[83] Almathami HKY, Than Win K, Vlahu-Gjorgievska E. Barriers and facilitators that influence Telemedicine-Based, Real-Time, online consultation at patients’ homes: systematic literature review. J Med Internet Res. 2020;22(2):e16407. 32130131 10.2196/16407 PMC7059083

[84] Ebert DD, Zarski A-C, Christensen H, Stikkelbroek Y, Cuijpers P, Berking M, et al. Internet and Computer-Based cognitive behavioral therapy for anxiety and depression in youth: A Meta-Analysis of randomized controlled outcome trials. PLoS ONE. 2015;10(3):e0119895. 25786025 10.1371/journal.pone.0119895 PMC4364968

[85] Regmi K, Jones L. A systematic review of the factors - Enablers and barriers - Affecting e-learning in health sciences education. BMC Med Educ. 2020;20(1):1–18. 10.1186/s12909-020-02007-6 PMC7106784 32228560

[86] Kauth MR, Shipherd JC, Lindsay JA, Kirsh S, Knapp H, Matza L. Teleconsultation and training of VHA providers on transgender care: implementation of a multisite hub system. Telemed e-Health. 2015;21(12):1012–8. 10.1089/tmj.2015.0010 26171641

[87] Shipherd JC, Kauth MR, Matza A. Nationwide interdisciplinary E-Consultation on transgender care in the veterans health administration. Telemed e-health. 2016;22(12):1008–12. 10.1089/tmj.2016.0013 27159795

[88] Blosnich JR, Rodriguez KL, Hruska KL, Kavalieratos D, Gordon AJ, Matza A, et al. Utilization of the veterans affairs’ transgender E-consultation program by health care providers: Mixed-Methods study. JMIR Med Inf. 2019;7(1):e11695. 10.2196/11695 PMC6682290 31344672

[89] Pratt-Chapman ML, Goltz H, Latini D, Goeren W, Suarez R, Zhang Y, et al. Affirming care for sexual and gender minority prostate cancer survivors: results from an online training. J Cancer Educ. 2020;37(4):1137–43. 33242160 10.1007/s13187-020-01930-y PMC8149486

[90] Costa AB, Pase PF, de Camargo ES, Guaranha C, Caetano AH, Kveller D, et al. Effectiveness of a multidimensional web-based intervention program to change Brazilian health practitioners’ attitudes toward the lesbian, gay, bisexual and transgender population. J Health Psychol. 2016;21(3):356–68. 26987830 10.1177/1359105316628748

[91] Barrett DL, Supapannachart KJ, Caleon RL, Ragmanauskaite L, McCleskey P, Yeung H. Interactive session for residents and medical students on dermatologic care for Lesbian, Gay, Bisexual, Transgender, and Queer patients. MedEdPORTAL. 2021;17:11148. 33907709 10.15766/mep_2374-8265.11148 PMC8063631

[92] Seay J, Hicks A, Markham MJ, Schlumbrecht M, Bowman-Curci M, Woodard J, et al. Web-based LGBT cultural competency training intervention for oncologists: pilot study results. Cancer. 2020;126(1):112–20. 31524952 10.1002/cncr.32491

[93] Enam A, Torres-Bonilla J, Eriksson H. Evidence-based evaluation of ehealth interventions: systematic literature review. J Med Internet Res. 2018;20(11):e10971. 30470678 10.2196/10971 PMC6286426

