# Supplementary material for: Enabling and hindering aspects of the i²TransHealth e-health intervention for transgender and gender diverse people in Germany: a qualitative process evaluation
Source: BMC Health Serv Res. 2026 Jan 24;26:149. doi: 10.1186/s12913-026-14026-y (PMC12849576; doi:10.1186/s12913-026-14026-y)
Supplement: Supplementary file 1 — Supplementary Material 1 [file 12913_2026_14026_MOESM1_ESM.docx]

**INTERVIEW GUIDELINE FOR NETWORK PHYSICIANS**

**Topic A: Open Introduction**

**Guiding Questions**

**Question 1:**

We would like to ask you about your experiences with our e-health intervention i²TransHealth. How would you assess your experiences? What was good, and what was less good? Everything that is important to you is important to us.

**Topic B: Specific Care Experiences**

**Guiding Questions**

**Question 1:**

I would like to specifically discuss the target group of i²TransHealth again. What experiences have you had with trans people in recent months?

**Question 2:**

Thinking about the study participants you have met, how severely were trans service users affected health-wise?

**Question 3:**

How did you perceive your medical role in relation to trans service users?

Please share your experiences from your professional perspective.

**Question 4:**

As part of your participation in i²TransHealth, have you changed anything in your medical approach to trans people or your practice? If so, what?

**Topic C: Experiences with the i²TransHealth Network and Infrastructure**

**Guiding Questions**

**Question 1:**

How have you experienced the training and exchange within the i²TransHealth network so far?

**Question 2:**

Has the project led to an additional workload in your practice?

**Question 3:**

How have you used the e-health platform?

**Topic D: Special Events**

**Guiding Questions**

**Question 1:**

Regarding the e-health intervention: What stands out in your memory the most?

**INTERVIEW GUIDELINE FOR SERVICE USERS**

**Topic A: Open Introduction**

**Guiding Questions**

**Question 1:**

We would like to ask you about your experiences with our e-health intervention i²TransHealth. How would you assess your experiences? What was good, and what was less good? Everything that is important to you is important to us.

**Question 2:**

i²TransHealth aims to improve access to informed healthcare for trans people in remote areas.

Has your participation in i²TransHealth changed anything in your life situation?
If so, in what way?

**Topic B: Specific Care Experiences**

**Guiding Questions**

**Question 1:**

What experiences have you had with your study therapists?

**Question 2:**

How did you experience the video consultation format?

**Question 3:**

Has the video consultation ever led to an additional burden in your daily life?

**Topic C: Experiences with the i²TransHealth Network and Infrastructure**

**Guiding Questions**

**Question 1:**

Have you made use of the local physicians’ network?

If so, what experiences have you had with it?

**Question 2:**

If you did not use the network, what were your main reasons for that?

**Question 3:**

How have you used the e-health platform?

**Topic D: Special Events**

**Guiding Questions**

**Question 1:**

Regarding the e-health intervention: What stands out in your memory the most?

**INTERVIEW GUIDELINE FOR STUDY THERAPISTS**

**Topic A: Open Introduction**

**Guiding Questions**

**Question 1:**

We would like to ask you about your experiences with the e-health intervention i²TransHealth. How would you assess your experiences? What was good, and what was less good? Everything that is important to you is important to us.

**Question 2:**

If i²TransHealth were to be permanently integrated into healthcare for trans people, what should be maintained, and what would need to change?

**Topic B: Specific Care Experiences**

**Guiding Questions**

**Question 1:**

I would like to specifically discuss the target group of i²TransHealth again. What experiences have you had with providing video-based mental health support for trans people through i²TransHealth?

**Question 2:**

How has the video consultation format influenced your psychotherapeutic work?

**Question 3:**

If the video consultation has caused a burden in your work, what contributed to this burden?

**Topic C: Experiences with the i²TransHealth Network and Infrastructure**

**Guiding Questions**

**Question 1:**

How has collaboration with the physicians' network worked?

**Question 2:**

How have you experienced the network of cooperating physicians?

**Question 3:**

How have you used the e-health platform for communication with service users?

**Topic D: Special Events**

**Guiding Questions**

**Question 1:**

Regarding the e-health intervention: What stands out in your memory the most?
